# Role of microRNAs in the Development of Cardiovascular Disease in Systemic Autoimmune Disorders

**DOI:** 10.3390/ijms21062012

**Published:** 2020-03-16

**Authors:** Chary Lopez-Pedrera, Nuria Barbarroja, Alejandra Mª Patiño-Trives, Maria Luque-Tévar, Carmen Torres-Granados, Mª Angeles Aguirre-Zamorano, Eduardo Collantes-Estevez, Carlos Pérez-Sánchez

**Affiliations:** Rheumatology Service, Reina Sofia Hospital/Maimonides Institute for Research in Biomedicine of Cordoba (IMIBIC)/University of Cordoba, E-14004 Córdoba, Spain; nuria.barbarroja.exts@juntadeandalucia.es (N.B.); alejandramaria.patino@gmail.com (A.M.P.-T.); merluqtev@gmail.com (M.L.-T.); carmenq.es@gmail.com (C.T.-G.); maaguirrezamorano@yahoo.es (M.A.A.-Z.); educollantes@yahoo.es (E.C.-E.); b32pesac@uco.es (C.P.-S.)

**Keywords:** systemic autoimmune diseases, antiphospholipid syndrome, systemic lupus erythematosus, rheumatoid arthritis, atherosclerosis, thrombosis, cardiovascular diasease, microRNAs

## Abstract

Rheumatoid Arthritis (RA), Systemic lupus erythematosus (SLE) and antiphospholipid syndrome (APS) are the systemic autoimmune diseases (SADs) most associated with an increased risk of developing cardiovascular (CV) events. Cardiovascular disease (CVD) in SADs results from a complex interaction between traditional CV-risk factors, immune deregulation and disease activity. Oxidative stress, dyslipidemia, endothelial dysfunction, inflammatory/prothrombotic mediators (cytokines/chemokines, adipokines, proteases, adhesion-receptors, NETosis-derived-products, and intracellular-signaling molecules) have been implicated in these vascular pathologies. Genetic and genomic analyses further allowed the identification of signatures explaining the pro-atherothrombotic profiles in RA, SLE and APS. However, gene modulation has left significant gaps in our understanding of CV co-morbidities in SADs. MicroRNAs (miRNAs) are emerging as key post-transcriptional regulators of a suite of signaling pathways and pathophysiological effects. Abnormalities in high number of miRNA and their associated functions have been described in several SADs, suggesting their involvement in the development of atherosclerosis and thrombosis in the setting of RA, SLE and APS. This review focusses on recent insights into the potential role of miRNAs both, as clinical biomarkers of atherosclerosis and thrombosis in SADs, and as therapeutic targets in the regulation of the most influential processes that govern those disorders, highlighting the potential diagnostic and therapeutic properties of miRNAs in the management of CVD.

## 1. Introduction

Systemic autoimmune diseases (SADs) are a heterogeneous group of disorders characterized by humoral, cell-mediated immune responses against various self-constituents. It is widely-known that SADs are the result of interaction between predisposing genetic factors, deregulation of the immune system, and environmental triggering factors [1]. Several systemic autoimmune conditions, including rheumatoid arthritis (RA), systemic lupus erythematosus (SLE), and antiphospholipid syndrome (APS), are linked to enhanced atherosclerosis, and consequently higher cardiovascular morbidity and mortality rates.

Thrombosis mainly occurs in the blood vessels surrounding the heart as well as in the brain and in peripheral vessels, thus contributing to the development of cardiovascular disease (CVD). Atheromatous plaque, which also primes thrombotic events, also develops in multiple places around peripheral blood circulation. The development of the distinct manifestations of CVD (including thrombosis, stroke of myocardial infarction, among others) has been demonstrated to derive from common pathological mechanisms [2]. In the setting of SADs, it has been demonstrated that the development of CVD involves several predisposing genetic elements, along with traditional modifiable risk factors (such as hypertension, obesity or hyperlipidemia), autoimmune mediators (i.e., autoantibodies and immune cells) and a number of inflammatory molecules [3,4,5,6,7].

From the genetic standpoint, it is known that in SADs there is a multifaceted interaction among various gene-products and genomic analyses (including gene-arrays and new generation sequencing studies) have demonstrated specific gene alterations in different tissues and immune cells of SAD patients, among which a number of pro-inflammatory and prothrombotic mediators, cell surface receptors and intracellular pathways are further involved in the development of inflammatory and CV diseases [8,9,10,11,12,13,14,15,16,17].

Yet, the analysis of gene expression does not completely explain the origin and progression of CV co-morbidities demonstrated in SADs. Epigenetics, which is defined by the modifications that have occurred in DNA that influence the phenotype without altering the genotype, constitute new mechanisms underlying gene regulation. Interrelated post-transcriptional and epigenetic mechanisms recognized to be altered in CV and autoimmune disorders are histone modifications, DNA methylation changes and microRNA activity, all of which act jointly by altering gene and protein expression levels [18]. Epigenetics determines a range of processes that are critical in the development and outcome of inflammation, CVD and thrombosis, such as angiogenesis, shear stress and atherosclerosis. Moreover, the reversibility of epigenetic alterations renders them valuable therapeutic targets for personalized medicine [2].

MicroRNAs (miRNAs), small non-coding RNAs that regulate gene expression, are key post-transcriptional regulators of a suite of molecular signaling pathways and pathophysiological cellular effects. Besides, circulating miRNAs have been recognized as disease biomarkers for both, diagnosis and development of personalized therapies in multi-faceted diseases [19].

This review focusses on recent insights into the potential role of miRNAs both, as clinical biomarkers of atherosclerosis and thrombosis in autoimmune diseases, and as therapeutic targets in the regulation of the most influential processes that govern those disorders, highlighting the potential therapeutic and diagnostic properties of miRNAs in the management of CVD.

## 2. Atherosclerosis, Thrombosis and Cardiovascular Disease in Autoimmune Disorders

Rheumatoid Arthritis (RA) Systemic lupus erythematosus (SLE) and antiphospholipid syndrome (APS) are the systemic autoimmune diseases most directly associated with an increased risk of developing cardiovascular (CV) events.

CVD in these autoimmune disorders results from a complex interaction among conventional CV risk factors (i.e., hypertension, hypercholesterolemia diabetes mellitus), immune deregulation (involving auto-antibodies, autoantigens and autoreactive leukocytes) and disease activity.

**Figure 1:**
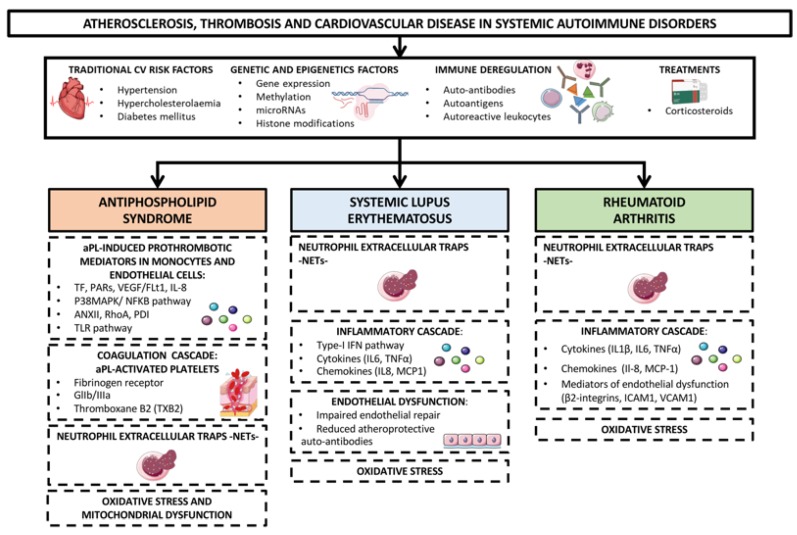
Mechanisms of atherosclerosis, thrombosis and cardiovascular disease in systemic autoimmune disorders. Rheumatoid arthritis (RA) Systemic lupus erythematosus (SLE) and antiphospholipid syndrome (APS) are the systemic autoimmune diseases most directly associated with an increased risk of developing cardiovascular (CV) events. CVD in these autoimmune disorders is thought to happen as the result of a complex interaction between traditional CV risk factors (i.e., hypertension, hypercholesterolaemia, diabetes mellitus), immune deregulation (involving auto-antibodies, autoantigens and autoreactive leukocytes), corticosteroids treatment and disease activity. In addition, oxidative stress and mitochondrial dysfunction, dyslipidemia, endothelial dysfunction, systemic inflammatory mediators -cytokines, chemokines, adipokines, proteases, adhesion receptors, products of NETosis, and intracellular signaling molecules- and prothrombotic molecules have been implicated in the development of these vascular pathologies. Genetic and epigenetic analyses have further allowed the identification of specific signatures explaining the pro-atherothrombotic profiles of RA, SLE and APS patients.

In addition, oxidative stress and mitochondrial dysfunction as well as dyslipidemia, endothelial impairment, systemic inflammation (promoted by cytokines, chemokines, adipokines, proteases, adhesion receptors, products of NETosis and a plethora of intracellular signaling molecules) and prothrombotic molecules, have been implicated in the development of these vascular pathologies [20,21]. Genetic and genomic analyses have further allowed the identification of specific signatures explaining the pro-atherothrombotic profiles of RA, SLE and APS patients (Figure 1) [22,23].

### 2.1. Antiphospholipid Syndrome

Antiphospholipid syndrome (APS) is an autoimmune thrombophilia, characterized by repetitive thromboembolism and/or pregnancy dismalness. It serological markers are the antiphospholipid antibodies (aPLs), mainly lupus anticoagulant (LA), anti-cardiolipin antibodies (aCL) and *anti*-β2 glycoprotein I (*anti*-β2GPI) which activate platelets, monocytes and endothelial cells, inducing the expression of tissue factor (TF, the main inductor of coagulation in vivo), protease activated receptors (PARs) and proinflammatory cytokines, -such as interleukins 6 (IL6) and 8 (IL8), vascular endothelial growth factor (VEGF) and its receptor 1, (Flt-1)-, a process that eventually leads to thrombus formation [24,25,26,27,28]. TF and VEGF expression are induced in monocytes and endothelial cells through the simultaneous and independent phosphorylation of mitogen-activated protein kinase/extracellular directed kinase protein (MEK-ERK42-44) and the p38 MAPK-dependent activation of NF-kappaB [29].

It has been also shown that aPLs promote an altered protein profile in monocytes related to thrombosis advancement, including overexpression of RhoA proteins, annexin I and annexin along with the reduced expression of protein disulfide isomerase (PDI) among others [30].

Furthermore, aPLs promote oxidative perturbations and mitochondrial dysfunction [31,32,33], which in turn generates an inflammatory profile, with expanded production of several cytokines, chemokines and promoters of endothelial damage [34,35].

Toll-like receptors 2 and 4 activate immune and endothelial cells, promote cytokine production, and favor pathogen recognition. In APS patients, mononuclear cells overexpress TLR2 and TLR4, which mediate aPL-induced vascular anomalies [36,37,38], pointing at these receptors as therapeutic targets to prevent aPLs-induced thrombosis in APS. Platelets can be also activated by aPLs, which induce fibrinogen receptor, thromboxane B2 and glycoprotein IIb/IIIa expression, supporting its aggregation and subsidizing thrombosis development [39].

Neutrophil extracellular traps (NETs) are also critical activators of the coagulation, and an essential component of blood vessels and venous thrombi. In that way, a number of studies by Knight and coworkers have determined that aPLs further activate neutrophils to release NETs, thereby predisposing to the arterial and venous thrombosis inherent in the APS [40,41,42].

Finally, numerous studies have illustrated that high aPL-IgG titers are strongly associated to the presence of atheroma plaques [33]. This aspect has been further confirmed by in vitro studies on which aPLs induced endothelial cells and leukocytes activation and foam cell generation by accelerating the adsorption by monocytes of oxidized low-density lipoproteins [43].

### 2.2. Systemic Lupus Erythematosus

Heightened rates of both cardiovascular events and prevalence of subclinical atherosclerosis, coronary artery calcification, endothelial dysfunction and aortic stiffness, have been demonstrated in patients with SLE. While, traditional CV factors, such as smoking, dyslipidemia, diabetes mellitus (DM), hypertension, central obesity and hyper-homocysteinemia have been reported to be prevalent in lupus patients, they do not fully explain the high rates of ischemic events and the enhanced risk of myocardial infarction and stroke that have been reported [44].

Several contributors, inherent to lupus itself, have been proposed to promote CVD. These contributors may include, among others [45,46]:

Clinical manifestations, such as the activity of the disease, leucopenia/lymphopenia or renal disease.

Prolonged treatments with corticosteroids or hydroxychloroquine, as well as cyclophosphamide or mycophenolate treatments.

Endothelial damage, promoted by deposition of oxLDL, autoantibodies against phospholipids and endothelial cells, type I IFNs and neutrophil extracellular traps -NETs-.

Presence of genetic variants in STAT4, interferon regulatory factor 8, mannose binding lectin, interleukin-19 and BAFF.

Inflammatory status, involving overexpression by monocytes and lymphocytes of chemokines and cytokines such as MCP-1, IL-6, IL-8 and TNFα.

Impaired atheroprotective mechanisms, such as decreased endothelial repair capacity and dampened production of atheroprotective autoantibodies.

The inflammatory burden and the excessive production of reactive oxygen species (ROS) by monocytes and neutrophils, further induced by most atherosclerosis risk factors, foster the development of the disease and enhance it CV risk [47].

### 2.3. Rheumatoid Arthritis

CVD is a frequent co-morbidity, present in RA patients. The development of premature atherosclerosis and CVD in these autoimmune patients involve several mechanisms, among which leukocytes play a key role. Autoimmune-mediated activation of leukocytes favor the production of inflammatory mediators, oxidative stress molecules and endothelial dysfunction, thus driving the development of atherosclerosis [48,49].

Monocytes infiltrate into joints, promoting inflammation, synovial proliferation and joint destruction in acute and chronic phases of RA [50]. Monocytes secrete pro-inflammatory cytokines, such as IL-1ß, IL-6, and TNFα, which in turn, promote endothelial activation/dysfunction [51,52]. Moreover, monocytes overexpress integrins (i.e., CD11c/CD18, CD11b/CD18), which facilitate their adhesion to the activated endothelium and their migration intro the arterial wall, where macrophages differentiate into foam cells, driving the development of atherosclerosis [53].

Recently, it has been demonstrated that neutrophils, that are well established players in host defense and acute inflammation, can also impact several clinical and immune related functions, from leukocyte recruitment and T-cell regulation to thrombosis and autoimmunity [54]. Neutrophils have recently been revealed as proatherogenic cells, so that neutrophils extracellular traps (NETs), generated through the so far well-known process of NETosis, have been detected in atherosclerotic lesions in both mice and humans [21]. It has been shown that synovial fluid neutrophils from RA patients suffer NETosis, which potentiate the inflammatory response [55,56]. Moreover, NETosis-derived products in the serum of RA patients had a diagnostic potential. In a recent study, we found that both, NETosis and the elements linked to the extrusion of NETs, are enhanced in neutrophils from RA patients. A significant relationship between NETosis-derived products and autoimmune and clinical parameters (i.e., titers of autoantibodies and the presence of a pathological increase in the carotid intima media thickness), was demonstrated in our RA cohort, along with oxidative stress markers and inflammatory mediators related to CVD. Therefore, our study demonstrated that NETs are key players in the disease activity of RA as well as on the pathophysiology of its pro-atherothrombotic profile.

## 3. MicroRNAs: Biogenesis and Mechanisms of Action

MiRNAs are a class of short non-coding RNAs of 18-22 nucleotides in length that regulate gene expression at the post-transcriptional level. They play a central role in the control of several pathways and biological processes, such as differentiation, development, apoptosis and survival [57]. MiRNAs have been also shown to be key players in the regulation of the innate and adaptive immune response [58]. Their altered expression and function have been linked to multiple disorders, including systemic autoimmune diseases [59,60] and cardiovascular pathologies [61,62].

### 3.1. MicroRNA Biogenesis

Human miRNAs are mainly encoded as individual genes (monocistronic) or in introns of host genes (mirtrons). However, they can also be found as long transcripts called clusters (polycistronic). The miRNA biogenesis is divided into canonical and non-canonical pathways. The canonical miRNA biogenesis starts with the transcription of a long hairpin primary miRNA (pri-miRNA) by the RNA polymerase II/III. The pri-miRNA is cleaved by the Microprocessor complex integrated by the RNase III enzyme Drosha and the RNA binding protein DiGeorge Syndrome Critical Region 8 (DGCR8), which generates a precursor miRNA (pre-miRNA). Once the pre-miRNA is generated in the nucleus, an Exportin 5 (XPO5)/RanGTP complex allows its export into the cytoplasm, where the RNase III enzyme Dicer cleaves it producing an intermediate miRNA duplex. This duplex is processed by the Argonaute (AGO) protein family, which load one strand of the mature miRNA in an RNA-induced silencing complex (RISC). The complex RISC, integrated by the mature miRNA, Ago, Dicer and a trans- activation-responsive RNA- binding protein (TRBP), finally facilitates the mRNA target recognition [63,64].

In addition to the canonical biogenesis pathway, different alternative routes have emerged in the last time. There are two main non-canonical pathways grouped into Dicer-independent and Drosha/DGCR8-independent pathways. Dicer-independent pathways include those short hairpin RNA transcripts, which are processed by Drosha and exported to the cytoplasm, are directly loaded into Ago-2 due to their insufficient length to be processed by Dicer [65,66]. Mirtrons are good examples of miRNAs processed via Drosha/DGCR-8-indepent pathway, since pre-miRNAs are directly generated by splicing and exported to the cytoplasm for Dicer processing [67,68]. In line with this, new examples have been also published such as the 7-methylguanosine (m7G)-capped pre-miRNA [69].

### 3.2. Mechanisms of Action of MicroRNAs

MiRNAs are mainly repressors of the gene expression, working in complex networks, since one miRNA can target several mRNAs and one mRNA can be controlled by different miRNAs [70]. Once the miRNA is loaded onto the RISC complex, the seed sequence of miRNA, which is located between nucleotides 2 and 7, is responsible for the mRNA target recognition in the 3′UTR region by sequence complementarity [71]. At early stages, miRNAs inhibit the translation of still stable mRNAs targets. However at large stages, miRNAs promote mRNA decay through mRNA deadenylation and decapping [72,73,74,75]. MiRNA and RISC complex promote the recruitment of poly(A)-binding proteins such as the deadenylase complexes poly(A)-specific ribonuclease 2 (PARN2), PARN3, carbon catabolite repressor 4 (CCR4) and negative regulator of transcription (NOT), which shorten the poly(A) tail of mRNAs. This process is also a signal for mRNA decapping through the recruitment of mRNA-decapping enzyme 2 (DCP2) along with stimulatory proteins, such as the DEAD box helicase DEAD box protein 6 (DDX6) which remove the 7-methylguanylate (m7G) cap [76,77,78,79].

While, the role of miRNA as negative regulators of gene expression has been fully characterized, new studies have also showed the capacity of miRNAs to activate gene expression. Various groups have shown that miRNAs can also bind 5’UTR, coding and promotor regions and induce the transcription, however, more studies are needed to fully characterize these new mechanisms of action [80,81,82,83,84].

## 4. Dysregulated Biogenesis of microRNAs in Systemic Autoimmune Diseases

The impact of the miRNA biogenesis machinery in the development of autoimmunity and CVD has been previously demonstrated, through the generation of several animal models, presenting deletions of Dicer, the master protein regulator of the miRNA biogenesis. In 2008, Zhou X et al., showed that a T-cell specific Dicer knockout mouse strain developed an uncontrolled autoimmunity through the disruption of the development and function of T reg cells. Dicer-deficient T reg cells showed a downregulation of the transcription factor FoxP3, along with an altered expression of multiple key gene and protein regulators of the T-cell function, which promoted the loss of their suppressor activity [85]. In line with these results, Muljo SA et al. also showed that the mouse deletion of Dicer in T-cells resulted in an aberrant differentiation of T-helper cells preferentially expressing a Th-1 phenotype with a remarkably production of IFN. These cells poorly proliferated upon stimulation and presented an increased apoptotic rate [86]. Regarding the role of Dicer in B-cells, Xu S et al. stated that the B-cell deletion of Dicer in mutant mice produced the upregulation of proapoptotic proteins and inhibitor genes of the cell cycle, resulting in defects in survival and B cell proliferation. Moreover, these cells also failed to produce high-affinity class-switched antibodies [87]. In parallel, another study, published by Koralov SB, indicated that the ablation of Dicer in early B-cells progenitors resulted in a developmental block at the pro- to pre-B cell transition and an impaired antibody diversification [88].

There are several evidences that miRNA biogenesis machinery has also a critical role in the development of the cardiovascular pathology. Thus, Wei Y et al., evidenced the central role of the miRNA biogenesis machinery in the development of atherosclerosis. The generation of Apoe-/- mice with macrophage-specific Dicer deletion accelerated the atherosclerotic process, increased lipid accumulation and enhanced inflammatory response. They concluded that Dicer plays an atheroprotective role with potential therapeutic implications to prevent this pathology [89]. Interestingly, another study also showed that the deletion of Dicer in the heart produced mice with heart failure, due to the drop in the expression of cardiac contractile proteins and profound sarcomere disarray [90]. Defects in Drosha have been linked to the development of cardiovascular disease. Thus, mutations in Drosha, the first enzyme of the biogenesis cascade, resulted in arteriovenous fistulae, hemorrhages, and disorganized and dilated vasculature [90]. In the same way, the inhibition of Dicer and Drosha with siRNAs in endothelial cells impaired angiogenesis, reducing tube forming activity and capillary sprouting [91].

The altered expression of the miRNA biogenesis machinery has been linked to several pathological conditions. Particularly in cancer multitude of studies have shown an aberrant expression of miRNA biogenesis proteins like Drosha, Dicer, DGCR8, Xpo-5, Ago-1 and Ago-2. Moreover, somatic and germline mutations have been associated with the miRNA biogenesis pathway in several cancers [92].

In the setting of SADs, our group have studied the role of the miRNA biogenesis machinery in three different diseases such as APS, SLE [93] and RA [94]. In a cohort of 23 APS and 64 SLE patients, and 56 healthy donors (HD) we showed that the mRNA levels of Dicer, Drosha, Ago-1, Ago-2 and Xpo-5 were reduced in neutrophils from APS and SLE patients in relation to HD. Moreover, the downregulation of Dicer, validated in both diseases at protein level, was accompanied by a global downregulation of the miRNA profile in neutrophils from SLE patients. In order to evaluate the clinical relevance of this miRNA biogenesis disruption in the development of the cardiovascular disease in APS and SLE patients, association studies with both, the presence of previous thrombotic events and the carotid intima media thickness (CIMT), as a marker of early atherosclerosis, were performed. In SLE patients the presence of previous thrombotic events was associated with reduced mRNA levels of Dicer, Drosha, Ago-1, Ago-2 and Xpo-5 in neutrophils. Furthermore, the downregulation of all these proteins was also significantly associated with an increased CIMT. In the case of APS patients, the presence of previous thrombotic events was associated with low levels of miRNA biogenesis proteins in neutrophils like Dicer, Ago-1, Ago- 2 and Xpo-5 but Drosha. Similarly, the downregulation of Dicer, Drosha and Xpo-5 was significantly linked to the presence of early atherosclerosis in these patients [93]. Along with neutrophils, the expression of miRNA biogenesis protein was also evaluated in other immune cells such as monocytes and lymphocytes; however, no changes were observed in APS and SLE patients compared with HD, highlighting the cell-specific alteration of this process.

The role of miRNA biogenesis in RA was also evaluated in other study in which we analyzed both the miRNA profile and miRNA biogenesis machinery in neutrophils from peripheral blood and synovial fluid in a cohort of 40 RA patients and 40 HDs. Neutrophils from the peripheral blood of RA patients displayed a downregulation of Dicer and Ago-1 in relation to HDs. Furthermore, the levels of Dicer, Ago-1, Ago-2, and Xpo-5 was also significantly reduced in neutrophils from synovial fluid of RA patients. This miRNA biogenesis disruption promoted the global downregulation of the miRNA profile in RA patients, being more accused in the case of neutrophils from synovial fluid.

The downregulation of both, the miRNA profile and miRNA biogenesis machinery, was associated with the disease activity (DAS-28, disease activity score), the autoimmune profile (anti-citrullinated protein antibodies (ACPA levels) and the expression levels of a number of inflammatory markers (TNFα, IL6, C-reactive protein). Moreover, with the aim of characterizing the impact of the downregulation of miRNA biogenesis machinery in neutrophils, in vitro experiments were carried out where Dicer was silenced in HL-60 neutrophil-like cells through lentiviral transfection. The reduction of Dicer provoked a global reduction of a key proinflammatory miRNAs, along with the upregulation of inflammatory and cardiovascular molecules such as PAI-1 (Plasminogen activator inhibitor-1), CCL-1, -2, -5, TNFα, MIP, MIF, IL-1, -8, -3, -16 and IL 17 [94].

## 5. Profile and Role of microRNAs in Systemic Autoimmune Diseases

MiRNAs regulate numerous physiological processes (i.e., cell metabolism, organ development, stem cell preservation). In autoimmune disorders such as APS, SLE and RA it have been also suggested pivotal roles for miRNAs in the modulation of a number of physio-pathological processes, such as angiogenesis, organ involvement and CVD [95]. However, the expression levels and function of miRNAs may by specific and distinctive for each autoimmune disorder.

### 5.1. Antiphospholipid Syndrome

The first miRNAs identified as involved in the pathogenesis of thrombosis in APS patients were miR-19b and miR-20a. Their levels were found significantly reduced in monocytes from APS patients in comparison with HD and negatively correlated with the overexpression of TF in these cells [96].

In a later study, we characterized a number of miRNAs related to the CVD present in APS patients, whose mains targets were linked to APS related features such as immune response, oxidative stress, atherosclerosis, and thrombosis: miR-125a-5p, miR125b-5p, miR-124a-3p, miR-146a-5p, miR-155-5p and miR-222-3p [93].The levels of these miRNAs were reduced in neutrophils from APS patients while in monocytes two of the miRNAs evaluated, miR-155 and miR-146a appeared increased. Altered levels of several miRNAs correlated with molecules related to oxidative stress, inflammation and thrombosis, and were linked to the presence of atheroma plaques (identified by increased carotid intima media thickness -CIMT- in Eco Doppler analyses) and to the occurrence of thrombotic events.

APS patients, with adverse pregnancy outcomes, also show distinctive and specific miRNA profiles. Thus, significantly higher levels of circulating exosome-associated miR-146a-3p, compared to healthy pregnant controls, has been identified. In addition, mechanistic studies have revealed that aPLs significantly induce trophoblasts to express higher levels of miR-146a-5p, miR-146a-3p, miR-155 and miR-210 [95]. These overall findings indicate that alterations in miRNA expression are closely related to the immunopathology of APS patients.

### 5.2. Systemic Lupus Erythematosus

Altered expression of miRNAs in mononuclear cells from peripheral blood, renal tissue and plasma from SLE patients have been demonstrated, implying their key role in the pathogenesis of this autoimmune disorder [97,98,99].

Dysregulated expression of miRNAs in immune cells from patients with lupus has been found mainly involved in their dysregulated inflammatory function, including T cell autoreactivity and cytokine production (mir-873, miR29b, miR21, miR31, miR145, miR224, miR125a, mir142-3p/5p, miR126, miR148a and miR155) [100,101,102,103,104,105,106,107,108,109], and B cell autoreactivity, proliferation and increased IgG production (miR1246, miR30a) [110,111], along with the regulation of NK cells (miR27a) [112].

Type I IFN pathway is recognized as the central detrimental factor the progression of SLE. Accordingly, there have been characterized several deregulated miRNAs in the immune cells of this patients as regulators of this pathway, including: miR-302d (downregulated in monocytes and involved in overactivation of the IFN pathway and the enhanced expression of the interferon regulatory factor-9 (IRF-9); miR-130b (downregulated in renal cells from lupus nephritis patients and acting as negative regulator of the type I IFN-pathway) [113]; miR-451 (whose silencing prime proteinuria and immune complex deposition in kidneys in vivo [114]).

More recently, it has been demonstrated lower miRNA expression of miR-361-5p, miR-128-3p and miR-181a-2-3p in plasmacytoid dendritic cells (pDCs) in SLE patients, also related to the IFN signature [115].

It has been demonstrated that whole peripheral blood miR146a and miR155 expression levels are increased in SLE patients and correlated with the diagnosis and the age of these patients [116].

The expression profile miRNAs is tissue-specific, so that the altered miRNAs in the kidney are absolutely distinct than that of peripheral blood in SLE patients [97,98].

Moreover, these patterns are closely related to the specific autoantibody conformation of different SLE patients. Furthermore, specific aberrant expression of miRNAs (especially hsa-miR-371-5p, hsa-miR-423-5p, hsa-miR-638, hsa-miR-1224-3p and hsa-miR-663) has been demonstrated in the PBMCs of lupus nephritis across patients with different races [117].

Circulating Serum and urinary miRNAs, presumed to be released from cells as a result of death, stress, or exocytosis, were shown further dysregulated and associated with different disease characteristics of SLE. Thus, in a recent study an deranged circulating miRNA profile was demonstrated in a wide cohort of SLE patients, among which a four-miRNA signature was identified as discriminative among SLE and HD, as well as to another set of miRNAs associated with LN [118]. More recently, an observational case-control cross-sectional study further identified several miRNAs, (including miR-221-5p, miR-380-3p, miR-556-5p, miR-758-3p and miR-3074-3p) as potential biomarkers of renal involvement in patients with SLE [119].

In sum, there are varieties of microRNAs identified as lupus biomarkers, including renal function-associated microRNAs, microRNAs involved in the immune system, and microRNAs for phenotype classification. Thus, miRNAs constitute a wide range of promising biomarkers aiding in the diagnosing and monitoring of lupus disease and the risk of organ damage.

### 5.3. Rheumatoid Arthritis

A number of dysregulated miRNAs in RA have been also recognized and further associated to the upregulation of several inflammatory cytokines and chemokines, along with inflammation-related signaling molecules, thus underlying their role in the pathogenesis of this autoimmune disorder [120]. In an excellent revision by Evangelatos et al., [121] a number of deregulated miRNAs in peripheral blood mononuclear cells (PBMCs) from RA were reported, which result in enhanced cytokine secretion and in disturbed Th17-Tregs balance in peripheral blood (miR-16, miR-103a, miR-132, miR-145, miR-146a, miR-155, miR-221, miR-222 and miR-301a were elevated; miR-21, miR-125b and miR- 548a were found decreased) [122,123,124,125,126,127,128,129,130,131,132,133,134,135,136].

Regarding T cells, miR-146a is increased in peripheral Th17 cells [137] and decreased in peripheral Tregs [138], both enhancing the secretion of proinflammatory cytokines. In addition, miR-223 is overexpressed in peripheral naïve CD4+ Τ cells and downregulated in Th17 cells [139].

Alterations in miRNA expression are also responsible for many aspects of rheumatoid arthritis synovial fibroblasts (RASFs) activity [140]. Most of the disturbed miRNA levels (mainly miR-124a, miR-126, miR-146a, miR-152, miR-155, and miR-221) [141,142,143,144,145,146,147,148,149,150,151,152,153,154,155] lead to the secretion of pro-inflammatory cytokines or matrix metalloproteinases (MMPs), as well as to the augmented survival and proliferation of RASFs.

In synovial tissue, altered levels of miR-27a, miR-30a, miR-708 and miR-206, have been found, mainly involved in the invasion of the cartilage by RASFs, enhanced autophagy of RASFs and synovial macrophages, increased RASFs survival and migration, and neovascularization [156,157,158,159]. Besides, altered expression of miR-let-7b, miR-155 and miR-223 in macrophages invading the synovial tissue, influence the development and perpetuation of synovial inflammation.

Additionally, some changes in circulating miRNAs have been identified as potential biomarkers for RA diagnosis. Thus, miR-24, miR-125a and miR-155, found increased in the peripheral blood, might be used to diagnose RA.

Many studies have further reported that altered levels of serum or PBMCs of several miRNAs might reflect disease activity [123,127,160], the status of activity or remission [161] and the relapse [162].

As for extra-articular manifestations, a few studies have reported the specific alteration of serum miRNAs in patients with interstitial lung disease, compared to RA patients without lung involvement [163], as well as among RA patients with or without peripheral neuropathy [164].

Finally, serum levels of miR-16, miR- 22, miR-23, miR-27, miR-103, miR-125b, miR-194, miR-210, miR-223, miR-432 and miR-886 has been demonstrated to be either, potential biomarkers of active disease, predictors of RA development in individuals with positive ACPAs, and/or predictors of treatment response [122,161,165].

## 6. Involvement of Auto-Antibodies on microRNA Biogenesis and Expression in Systemic Autoimmune Diseases

A number of studies have described the in vivo correlation between the expression level of dysregulated miRNAs (and their biogenesis proteins) in APS, SLE and RA, and the levels of specific autoantibodies present in each autoimmune disorder (aPLs, dsDNA, ACPAs, etc.) [93,94,166,167].

In line with this fact, our group have conducted in vitro studies to analyze the involvement of specific autoantibodies in the expression of several miRNAs and their biogenesis machinery in both, immune and endothelial cells. We demonstrated that the treatment of endothelial cells and neutrophils from HDs with either, aPL isolated from the serum of APS patients or anti-ds-DNA antibodies isolated from the serum of SLE patients, promoted the downregulation of Dicer (both, mRNA and protein levels) along with that of a set of miRNAs previously characterized as key regulators of proteins involved in the atherothrombotic process in these patients (miR-124a, -125a, -125b, -222, -146a, -155). Accordingly, when these experiments were carried out on monocytes, the expression of miR-146a and miR-155 were upregulated, while miR-124a was downregulated, resembling the altered patterns that these patients exhibited in vivo [93]. As in the case of aPLs and anti-ds-DNA antibodies, ACPAs isolated from the serum of RA patients induced on healthy neutrophils the downregulation of Dicer, Ago-2 and Xpo-5, as well as a set of microRNAs (miR-let7b, -126, -148a, -29c, -17, -21, -223) regulators of targets involved in cell migration, inflammation and cell survival, which were upregulated in a parallel manner [94].

The aPL antibodies have also shown the capacity to promote the secretion of miRNAs. Thus, Gysler SM et al. showed that the treatment of trophoblast cell line with anti-β2GPI antibodies promoted an increased secretion of miR-146a-5p, miR-146a-3p, miR-155, and miR-210 to the supernatant [168]. Similarly, we have demonstrated that the treatment of endothelial cells, with aPLs-induced changes in the expression of secreted miRNAs that had been previously shown to be part of the dysregulated signature of miRNAs in the plasma APS patients [166]. In another study published by Wu M et al., the treatment of endothelial cells with anti-β2GPI antibodies, produced the secretion of extracellular vesicles with a different content of miRNAs compared to the treatment with non-immune Ig-G [169]. Thus, the aPLs seems to play a role not only in the activation of intracellular targets through the control of the expression of specific miRNAs, but also in the secretion of mediators that presumably might have an impact in other cell types involved in the pathogenesis of APS patients.

## 7. Cellular microRNAS as Biomarkers of Cardiovascular Disease in Systemic Autoimmune Disorders

Autoimmune diseases (AIDs) have been associated with accelerated atherosclerosis (AT), leading to increased cardio- and cerebrovascular disease risk. Inflammation and atherosclerosis have been demonstrated to be intimately connected, so that a number of inflammatory-related processes, such as endothelial dysfunction, oxidative stress, macrophage increase, toll-like receptor signaling, and cytokine secretion are some of the mechanisms implicated in the atherogenic process.

There is growing evidence that miRNAs control these pathologic processes including endothelial dysfunction (miR let-7g, mir-17-3p, miR-31, miR-146a, miR-155, miR-181 family, miR-221/-222,) [170,171,172,173,174,175], oxidative stress (miR-let-7a/b, miR-19b, miR-20a, miR-98, miR-126, miR-142-3p, miR-199a-3p and -5p, miR-200c, miR-221, miR-222, miR-328) [176,177,178,179,180,181,182,183,184,185,186,187,188,189], monocyte recruitment, differentiation and activation (miR-21, miR-23a-5p, miR-27a/b, miR-33, miR-34, miR-146a, miR-155, miR-212, miR-451, miR-590, miR-758-5p, [190,191,192,193,194,195,196,197,198,199,200,201] and inflammation/secretion of inflammatory cytokines (miR let-7g, miR-17-3p, miR-31, miR-146a, miR-155, miR-181a-3p/-5p, miR-181b, miR-221 and miR-222) [171,202,203]. Interestingly, a number of them are simultaneously involved in all those processes, suggesting common and/or cooperative activities, and underlying their relevance in the physiopathology of CVD.

**Figure 2:**
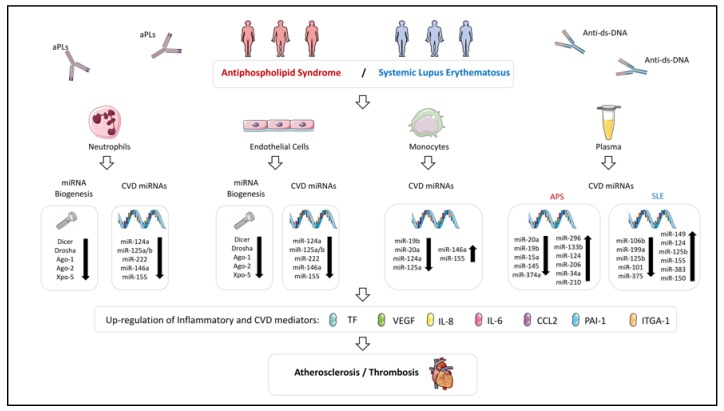
Altered expression of miRNAs and their biogenesis machinery in immune cells influencing both atherosclerosis and thrombosis processes in Antiphospholipid Syndrome and Systemic Lupus Erythematosus. miRNA expression profile is dysregulated in immune cells and plasma of APS and SLE patients. Those altered miRNAs could modulate the expression of inflammatory and CVD mediators, being associated with clinical pathological features of these diseases, such as atherosclerosis and thrombosis. The key role of the autoantibodies present in these diseases in the alteration of the miRNA profile has been confirmed indeed through in vitro studies.

**Figure 3:**
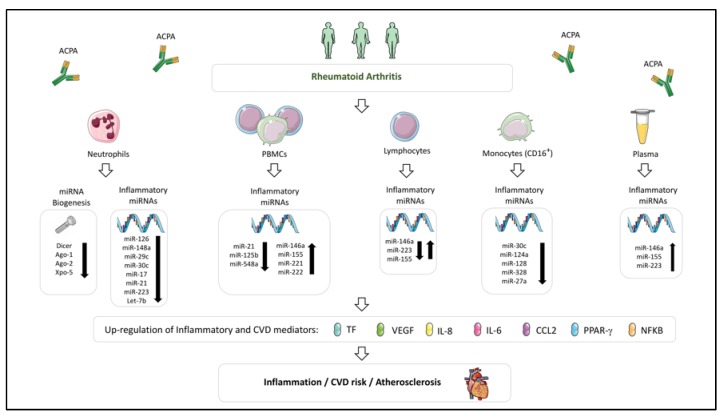
Dysregulated miRNA profile involved in the development of CVD in Rheumatoid Arthritis. Altered levels of miRNAs have widely been described in immune cells (neutrophils, PBMCs, lymphocytes and monocytes) and plasma of rheumatoid arthritis patients. This dysregulation would modulate the expression of inflammatory and CVD mediators, favoring the development of atherosclerosis and CVD. In these miRNA alterations, a relevant role has been attributed to the presence of anti-citrullinated protein antibodies (ACPAs).

Accordingly, abnormalities in a high number of those miRNA and their associated functions have been described in several SADs, pointing at their involvement in the development of atherosclerosis and thrombosis in the settings of RA, SLE and APS (Figure 2 and Figure 3).

### 7.1. Cellular microRNAs Related to CVD in Systemic Lupus Erythematosus and Antiphospholipid Syndrome

In SLE, relevant factors directly influencing the development of atherosclerosis and CVD include immune-complex formation, complement activation and altered expression and activity of numerous cytokines and chemokines (i.e., B lymphocyte stimulator -BLyS-, type I and II interferons, migration macrophage inhibitor -MIF-, IL-6, IL-17 and TNFα [7,204,205,206,207,208,209,210,211,212,213,214]).

The expression shape of miRNAs in blood cells, tissues and fluids of SLE patients has been extensively analyzed. Hence, deregulated miRNAs contributing to SLE pathogenesis have been identified, including various regulating DNA methylation of T lymphocytes, type I interferon pathway, and local tissue inflammation (i.e., miR-15, miR-21, miR-31, miR-125a, miR142, miR-146a, miR-155, and miR-181 [215]). Moreover, their expression has been demonstrated to influence several parameters related to disease activity and organ damage [216,217]. However, to date, only a few studies have characterized miRNAs, related to the CV and atherothrombotic risks, observed in APS and SLE.

Recently, we developed a work with the aim of identifying and characterizing miRNAs related to the pathogenesis of CVD in APS and SLE patients [93].

All the miRNAs analyzed were found reduced in neutrophils both, APS and SLE patients, in parallel to a significant decrease in molecules related to miRNA biogenesis (i.e., Dicer, Drosha, Exportin-5, Argonaute-1 and -2). Conversely, in monocytes, increased expression of miR-146a and miR-155 were shown, which positively correlated with levels of specific APS and SLE autoantibodies (ACA-IgG and anti-dsDNA antibodies, respectively), along with plasma levels of oxidative stress molecules and inflammatory mediators. Consistent with previous studies, that data suggests that monocyte overexpression of both miRNAs might act as protective mechanisms against the pro-inflammatory effects of autoantibodies [103,128,150,218,219].

On the contrary, reduced levels of miR-125a (involved in the activation of the proinflammatory pathway as well as in the increased expression of RANTES) were found in both, neutrophils and monocytes from APS and SLE patients, and negatively correlated with prothrombotic, proinflammatory and oxidative stress mediators. Moreover, reduced miR-125a levels were associated to the incidence of thrombosis in these autoimmune conditions [106].

Also, miR-124a (which targets monocyte chemoattractant protein 1 (MCP-1) [143]), was found reduced in vivo in monocytes and neutrophils from APS and SLE patients, modulated in vitro by the autoantibodies from both autoimmune conditions, and related to the presence of a pathological increase of CIMT and to the occurrence of thrombotic events, as previously reported in other autoimmune conditions [33,220,221]. Thus, both, miR124a and miR-125a, seem to act a key players in the development of CVD in APS and SLE.

Our study reported an altered expression of several miRNAs in APS and SLE patients linked to the pro-oxidative status and the mitochondrial dysfunction present in both diseases (i.e., miR-125a, miR-146a and miR-155), and thus, involved in inflammation and CVD [222,223,224]. Hence, the levels of these miRNAs correlated with the augmented percentage of cells, with altered ΔΨm, and the increased activity of mitochondrial enzyme superoxide dismutase.

It is well-known that miRNAs expressed in the vasculature play important roles in CVD [225], so that a number of them are overexpressed in inflamed ECs. Thus, it has been shown that TNFα downregulates miR-181b expression (which promoted increased expression of adhesion molecules such as VCAM-1) [226] and induce miR-17, miR-31, miR-155, miR-221 and miR-222 (provoking neutrophil and T cell adhesion of ECs, along with proliferation and migration of ECs) [173,174]. In line with these studies, our study demonstrated that specific autoantibodies from APS and SLE patients (aPL-IgG and anti-ds-DNA-IgG, respectively) downregulated in ECs all the miRNAs evaluated and altered the expression of several markers of endothelial dysfunction, such as TF, VCAM-1 and eNOS. Therefore, that miRNAs seem to further regulate the endothelial dysfunction observed in these autoimmune diseases.

### 7.2. Cellular microRNAs Related to CVD in Rheumatoid Arthritis

In RA, the expression levels of several miRNAs by immune cells have been found associated with inflammation and cytokine production, and some of them correlated with disease activity and enhanced CV risk [227].

Extensive studies have highlighted the key role of monocytes in the pathogenesis of RA, involving both, the infiltration into joints, which promote inflammation of the synovium and joint destruction [50], and the secretion of inflammatory cytokines into the systemic circulation -which provoke endothelial dysfunction and atherosclerosis development [51,52]-.

Human monocytes comprise two subpopulations, the classical (CD14+ + CD16−) monocytes (up to 90% of blood monocytes), and CD16+ monocytes, which are further divided in two subgroups: Intermediate (CD14^high^CD16^low^) and non-classical (CD14^low^CD16^high^) monocytes. The “intermediate” monocyte subpopulation secretes proinflammatory cytokines, such as TNFα and IL1ß and has been shown to be involved in inflammatory immune responses [228], while “non-classical” monocytes display high migratory, but reduced phagocytic potential [229]. Increased frequency of CD16+ monocytes has been reported in patients with active RA [230].

In that way, in a very recent study, we developed a molecular characterization of monocyte subsets from RA patients that revealed specific and distinctive signatures associated with CVD [231].

That study demonstrated a differential and specific miRNA profile in CD16+ monocytes in comparison to CD14+ monocytes, involving a higher number of deregulated miRNAs with potential targets related to thrombosis, inflammation and immunity, and linked to the presence of atheroma plaques. Specifically, CD16+ monocytes displayed reduced expression of several miRNAs (miR-30c-5p, miR-124a-3p, miR-128-3p, and miR-328-3p) that paralleled the increased mRNA expression levels of IFNγ, IL-6, PPARγ, and TF. Conversely, reduced expression of miR27a-3p in CD14+ monocytes was associated with the presence of early atherosclerosis, which paralleled the increase in IL-8, MCP-1, VEGF, and TF. Our overall data suggested that the altered expression of a number of miRNAs in CD14+ and CD16+ monocytes play an essential role in the development of the endothelial dysfunction present in RA patients, which in turn, heralds the progression of atherosclerosis and drives the development of CVD.

Altered expression of the above-mentioned atherosclerosis and CVD-related miRNAs, along with other miRNAs recognized as regulators of inflammatory pathways, has been also demonstrated in RA patients and might thus be further associated to CVD.

One of the most widely studied is miR-146a-5p, which is increased in many cell types in RA such as synovial fibroblasts, peripheral blood mononuclear cells, CD4+ T cells, and Th17 cells, and acts as a negative regulator of NFκB activation [232]. Li and colleagues [233] showed that miR-146a expression in CD4+ T cells from synovial fluid and peripheral blood of RA patients was significantly up-regulated and positively correlated with levels of tumor necrosis factor-alpha (TNFα). Niimoto and coworkers [137] demonstrated the relationship among the expression of miR-146a and that of IL-17 in PBMCs and synovium of RA patients.

Another miRNA found altered in RA patients and closely related to their inflammatory profile is miR-155-5p, a major regulator of B cell development and function, T cell dependent antibody responses, and T cell functions, including the overexpression of proinflammatory cytokines, such as TNFα and IL-1. MiR-155 is increased in the sera of RA patients [160], as well as in macrophages inside the synovium of RA patients, thereby enhancing their survival and cytokine production, [126] and correlated with high disease activity (DAS28) [127]. Moreover, genetic silencing of miR-155 gene led to resistance to arthritis development [130].

MiR-221 and miR-222 levels in PBMCs have been found analogous to disease activity [132] and involved in osteoblastogenesis [234] and the increased production of pro-inflammatory cytokines [149].

miR-223-3p modifies inflammation, so that it has been demonstrated that its levels are increased in synovial macrophages of RA patients [235,236] and that its overexpression leads to increased pro-inflammatory cytokines secretion.

In addition to their association with disease activity and inflammation in the setting of RA, some of these miRNAs have a clear relationship to cardiovascular disease. Thus, coronary syndrome patients have been demonstrated to display increased miR-146 expression [237] in the plasma and peripheral blood mononuclear cells. Besides, it has been demonstrated that miR-146a-5p affects endothelial function by regulation of nitric oxide production [238]. Moreover, the over-expression of miR-146a was found to significantly upregulate the function of Th1 cells. Furthermore, miR-146a treatment in vitro may induce the expression by these cells of key inflammatory cytokines/transcription factors involved in atherosclerosis (i.e., TNFα, MCP-1 and NFκB).

MiR-221 and miR-222, which are significantly increased in the intima of atherosclerotic lesions, promote endothelial dysfunction through the increase of reactive oxygen species production. These miRNAs also promote proliferation in VSMCs and influence the angiogenic capacity of ECs [239] and phenotypic properties of VSMCs [240]. Moreover, they have been found also elevated in the serum of patients with clinical atherosclerosis and correlated with triglyceride and VLDL levels [241].

Accordingly, miR-223-3p decreases cholesterol biosynthesis and enhances cholesterol efflux [242]. Moreover, delivery of miR-223-3p to endothelial cells decreased adhesion molecule expression [243], a key factor in the development of atherosclerosis.

While confirming studies are still required, that overall data support that miR-146a, miR-155, miR-221, miR-222 and miR-223 might be involved in the pathogenesis of CVD in the setting of RA.

## 8. Circulating miRNAs as Biomarkers of CV Disease in SADs

MiRNAs can be detected in different biological fluids including plasma, serum, saliva, semen, tears, saliva, urine and cerebrospinal fluid [244,245,246,247]. They are actively secreted by cells and tissues and seem to play a role in cell-to cell communication [248]. In circulation, they are protected from ribonucleases in microvesicles (exosomes, apoptotic bodies, microparticles), in protein complexes (Ago2, NPM1) and in lipoprotein complexes (LDL, HDL) [249]. Thus, circulating miRNAs have been pointed as ideal candidates as biomarkers of disease, and nowadays, the number of studies analyzing their potential is increasing exponentially.

There are several studies highlighting the potential of circulating miRNAs as biomarker of disease in both, SADs and CVD diseases. In SLE, various groups have revealed specific signatures of circulating miRNAs that can differentiate patients from healthy donors [118]. They have also shown capacity to be considered as potential biomarkers of renal disease [119,250,251,252] and for monitoring the response to therapy [253]. In RA, the diagnostic potential of circulating miRNAs and their association with the disease activity has also been elucidated [254,255]. Furthermore, our group also described the role of serum miRNAs as a predictor of response to anti-TNFα therapy in RA patients [256]. In other pathological conditions, such as cardiovascular diseases, circulating miRNAs have been also shown promising results. Thus, in the two major causes of cardiovascular mortality such as heart failure and acute myocardial infarction, circulating miRNAs have shown to have the capacity to improve the accuracy of diagnosis and prognosis of other protein biomarkers [257,258].

The potential role of circulating miRNAs as biomarkers of CVD in SADs is still being analyzed. We recently studied the plasma miRNA profile in a cohort of 90 APS patients and 42 healthy donors [166]. We demonstrated a specific signature of 10 miRNA ratios integrated by 11 miRNAs (34a-5p, 15a-5p, 145a-5p, 133b-3p, 124-3p, 206, 20a-5p, 19b-3p, 210-3p, 296-5p and 374a-5p) that accuracy discriminated APS patients from HD (80% of specificity). The target prediction analysis showed that these miRNAs are potential modulators of key proteins involved in the thrombotic pathology of APS. The specificity of that signature in APS was confirmed after the comparison with two independents cohorts including 23 thrombotic patients, without autoimmune diseases, and 25 SLE patients, without aPLs. The signature of circulating miRNAs was also associated with clinical features of APS such as the presence of fetal loss and the type of thrombosis. Interestingly, the signature integrated by miR-19b, miR-124, and miR-296 identified with high accuracy APS patients with an increased CIMT, highlighting the potential role of these circulating miRNAs as biomarkers of early atherosclerosis in APS patients. Furthermore, a cluster analysis also showed that the signature of miRNAs was differentially expressed between groups of patients with different thrombotic risk profiles [166].

In SLE patients, Kay SD et al., investigated the association of circulating microRNAs with the cardiovascular disease presents in these patients. In a cohort of 121 SLE patients, the presence of atherosclerosis, by carotid ultrasound and computed tomography, was detected in 50 patients. In parallel, a panel of 46 selected miRNAs related to rheumatic and atherosclerotic disease, based on literature, was analyzed in all subjects. The reduction in the expression of three miRNAs (miR-125b, miR-101, and miR-375) was associated with the presence of atherosclerosis. The panel integrated for these miRNAs showed an area under the curve (AUC) of 0.7. Then, the combination of these miRNAs with clinical variables associated with atherosclerosis such as gender, hypertension and smoking status, through a multivariate logistic regression analysis, improved the accuracy of the model up to an AUC of 0.9 [259]. Another work published by Carlsen AL and colleagues identified that increased levels of miR-155, miR-383, and miR-150 were significantly associated with the presence of vascular events (arterial thrombosis, myocardial infarction, or venous thrombosis) in a cohort of 68 SLE patients [88] (31 presenting vascular events) [118].

Although no study has been developed to date to identify a complete profile of circulating miRNAs as potential biomarkers of CVD in the setting of RA, altered serum levels of several miRNAs known to be closely related to CV disorders, such as miR-146a, miR-155 or miR-223 have been identified in these patients, thus suggesting their potential role in that frequent comorbidity in RA patients.

Overall, these studies showed the potential of circulating miRNAs as biomarkers of CVD in SADs (Figure 2 and Figure 3). However, new studies, using bigger cohorts of patients analyzing the whole miRNome, and applying integrated bioinformatics approaches using other biomarkers, are still needed.

## 9. Role of miRNAs on the Response to Therapy

The aberrant expression of cellular and circulating miRNAs that regulate the expression of inflammatory and cardiovascular mediators in SADs have been found to be modified in response to different therapies. Thus, we showed that the supplementation with 200 mg/day of Ubiquinol (reduced form of CoQ10) for one month in a cohort of 36 APS patients, was able to reverse in monocytes the expression levels of a set of altered microRNAs (17-5p, 146b-5p, 29a-3p, 145-5p, 199a-5p, 494-3p, let-7f-5p). Moreover, this supplemental therapy reduced the expression of a number pro-atherothrombotic proteins modulated by these microRNAs (IL1A, IL6, VEGFA, TNFα, IFNG, IL8, SERPINB2, ITGA2, LPL and TF among others) [260]. In a recent study, we also showed that Rituximab (RTX) (375 mg/sq weekly for 4 weeks) reversed the altered signature of miRNAs in the plasma of SLE patients after 3 months of therapy. Thus, RTX restored the level of a panel of 5 miRNAs, including miR-106b-3p, 149-3p, 125b-5p, 199a-5p, and miR-124-3p, which showed potential targets molecules involved in the control of inflammation and vascular function (TNFα, TLR9, CD3E, CD3G, TNFRSD13C, CD40LG, FGFRs, HLA-C, IL1F10, HLA-A, IL1RN, IL6R, IL36B) [253]. In RA, the role of anti-TNF therapies as modulators of miRNA expression has been also analyzed. In a cohort of 90 RA patients the expression levels of miR-16-5p, miR-23-3p, miR125b-5p, miR-126-3p, miRN-146a-5p, miR-223-3p were up-regulated in the serum of these patients after 6 months of TNFα/DMARDs combination therapy. Target prediction analysis showed that these miRNAs might control the expression of mRNA targets, involved in immune and inflammatory response, cardiovascular system development and function, and connective tissue and musculoskeletal system. Moreover, several genes were potential targets of more than one of these miRNAs at the same time (CHUK, IL6R, IL6ST, FGF2, BMPRII). The upregulation of these miRNAs was significantly correlated with the reduction of the disease activity and inflammatory markers (ESR and CRP) [256]. Taken together, the therapeutic modulation of a complex network of miRNAs and targets might be a new strategy for preventing the CVD pathology of SADs patients.

## 10. Conclusions

In recent years, studies on miRNAs have updated our understanding of the etiology and pathogenesis of autoimmune diseases. It is now well-demonstrated that numerous miRNAs regulate the proliferation, differentiation and function of immune and vascular cells, thus playing critical roles in the pathophysiology of these disorders.

Moreover, miRNAs control post-transcriptional processes that regulate the expression of genes involved in the development of different but interrelated disorders, such as autoimmune and cardiovascular diseases. Hence, they might have a regulatory role in pathways shared by these diseases. Thus, the study of the miRNA profile might be useful to identify new biomarkers as potential predictors of the development of severe complications in these pathologies.

miRNAs seem to be important regulators of networks instead of single genes. Thus, their potential use as biomarkers should rely on a functional integrative analysis of their effects in the biological mechanisms underlying autoimmune diseases and their associated comorbidities, which until now, are missing. At the same time, the identification of specific altered miRNAs might allow the characterization of precise pathogenic pathways and suggest new treatment strategies.
